# In artemisinin-resistant falciparum malaria parasites, mitochondrial metabolic pathways are essential for survival but not those of apicoplast

**DOI:** 10.1016/j.ijpddr.2024.100565

**Published:** 2024-09-19

**Authors:** Manel Ouji, Thibaud Reyser, Yoshiki Yamaryo-Botté, Michel Nguyen, David Rengel, Axelle Dutreuil, Marlène Marcellin, Odile Burlet-Schiltz, Jean-Michel Augereau, Michael K. Riscoe, Lucie Paloque, Cyrille Botté, Françoise Benoit-Vical

**Affiliations:** a LCC-CNRS, Laboratoire de Chimie de Coordination, Université de Toulouse, CNRS, Toulouse, France; b MAAP, New Antimalarial Molecules and Pharmacological Approaches, Inserm ERL 1289, Toulouse, France; c Institut de Pharmacologie et de Biologie Structurale (IPBS), Université de Toulouse, CNRS, Université Toulouse III - Paul Sabatier (UT3), Toulouse, France; d ApicoLipid Team, Institute for Advanced Biosciences, CNRS UMR5309, Université Grenoble Alpes, INSERM U1209, Grenoble, France; e Infrastructure nationale de Protéomique, ProFI, FR 2048, Toulouse, France; f VA Portland Health Care System Research and Development Service, 3710 SW US Veterans Hospital Road, RD-33, Portland, OR, 97239, USA; g Department of Molecular Microbiology and Immunology, Oregon Health & Science University, 3181 SW Sam Jackson Park Road, Portland, OR, 97239, USA

**Keywords:** Artemisinin resistance, Metabolism, Apicoplast, Mitochondrion, Quiescence

## Abstract

Emergence and spread of parasite resistance to artemisinins, the first-line antimalarial therapy, threaten the malaria eradication policy. To identify therapeutic targets to eliminate artemisinin-resistant parasites, the functioning of the apicoplast and the mitochondrion was studied, focusing on the fatty acid synthesis type II (FASII) pathway in the apicoplast and the electron transfer chain in the mitochondrion. A significant enrichment of the FASII pathway among the up-regulated genes in artemisinin-resistant parasites under dihydroartemisinin treatment was found, in agreement with published transcriptomic data. However, using GC-MS analyzes of fatty acids, we demonstrated for the first time that the FASII pathway is non-functional, ruling out the use of FASII inhibitors to target artemisinin-resistant parasites. Conversely, by assessing the modulation of the oxygen consumption rate, we evidenced that mitochondrial respiration remains functional and flexible in artemisinin-resistant parasites and even at the quiescent stage. Two novel compounds targeting electron transport chain (ELQ300, ELQ400) efficiently killed quiescent artemisinin-resistant parasites. Therefore, mitochondrial respiration represents a key target for the elimination of artemisinin-resistant persistent *Plasmodium falciparum* parasites.

## Graphical abstract

**Figure:**
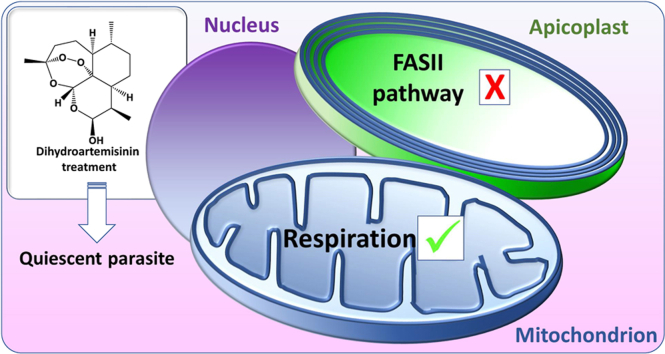

## 1 Introduction

**Scheme 1:**
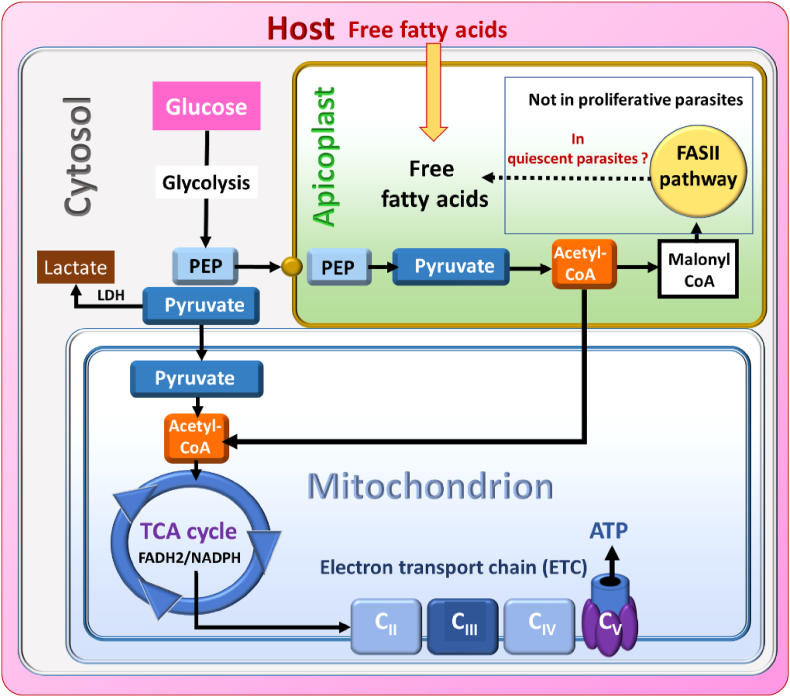
Synthetic model of *Plasmodium falciparum* metabolism. LDH: lactate dehydrogenase, PEP: phosphoenolpyruvate, TCA: tricarboxylic acid, C_II_-C_III_-C_IV_-C_V_: complexes of the electron transport chain.

Malaria remains one of the deadliest of infectious diseases worldwide despite countless efforts to control and eradicate it. In 2022, 249 million cases were reported resulting in 608,000 deaths mainly due to *Plasmodium falciparum* parasites (WHO, 2023). The recommended therapy is the use of Artemisinin-based combination therapies (ACT), combining artemisinin derivatives with another antimalarial drug (WHO, 2023). This strategy has been efficient on *Plasmodium* until the emergence and spread of parasites resistant to artemisinins (*i.e.* artemisinin and its derivatives; ART) in many areas (Dondorp et al., 2009; Mathieu et al., 2020; Uwimana et al., 2020; Balikagala et al., 2021; Fola et al., 2023). In addition, ART-resistant parasites have also become resistant to partner drugs in ACTs (Na-Bangchang et al., 2013; Duru et al., 2015; Siddiqui et al., 2021; Tumwebaze et al., 2022). Based on *in vitro* studies and *P. falciparum* infection models, it has been shown that in parasite populations exposed to ART, only a sub-population escapes drug exposure by entering in a quiescence/dormancy stage that corresponds to a cell cycle arrest which can be associated with an extended ring stage (Hoshen et al., 2000; Teuscher et al., 2010; Witkowski et al., 2010; Codd et al., 2011; Chen et al., 2014; Peatey et al., 2015, 2021; Intharabut et al., 2019; Connelly et al., 2021; Auparakkitanon and Wilairat, 2023). Upon elimination of ART treatment, surviving parasites are able to resume a normal cell cycle and proliferate leading to a persistent parasitemia in patients, at the end of the treatment, and an extended half-life of the parasite clearance (Dondorp et al., 2009; Ward et al., 2022). Resistance to ART, mainly linked to *pfk13* gene polymorphism (Ariey et al., 2014; Straimer et al., 2015), appeared to be multifactorial with an increased production of PI3P (Mbengue et al., 2015), the activation of the unfolded protein response (Mok et al., 2015), the phosphorylation of the eukaryotic transcription factor eIF2α (Zhang et al., 2017), the adjustment of the central carbon-linked pyruvate and glutamate metabolism at the mitochondrial level (Mok et al., 2021), a reduced endocytosis of hemoglobin at the ring stage in mutated parasites, leading to a reduced oxidative stress generated by artemisinin (Yang et al., 2019; Birnbaum et al., 2020), and an enhanced capacity to handle oxidative stress with a more efficient redox system compared to sensitive parasites (Egwu et al., 2022). All these metabolic and biochemical modifications appear to be necessary to trigger a cell cycle arrest during ART treatment (Keroack and Duraisingh, 2022). The first transcriptomic studies showed down-regulation of most of the parasite metabolic pathways including folate and isoprenoid metabolism, glycolysis, RNA and DNA synthesis, as well as up-regulation of pathways in apicoplast triggering type II fatty acids (FASII) synthesis, and the maintenance of a functional respiratory chain in mitochondrion (Chen et al., 2014; Connelly et al., 2021; Mok et al., 2021; Peatey et al., 2021). Overall, these data suggested a role for the apicoplast and mitochondrion in parasite quiescence. The FASII pathway is based on the import in the apicoplast of phosphoenolpyruvate (PEP) and acetyl-CoA, both derived from parasite glycolysis (Scheme 1), the main source of energy for *P. falciparum* during the erythrocytic stage (Cobbold and McConville, 2014). The FASII pathway also relies on the core activity of the ACP (Acyl Carrier Protein), which brings fatty acids to the catalytic site of each of the four core enzymes of this pathway (Waller RF et al., 1998). Since the disruption of the gene coding for a FASII enzyme (Fab I) did not affect *P. falciparum* asexual blood stage growth, FASII was assumed to be dispensable at this stage (van Schaijk et al., 2014) as parasites scavenge the necessary fatty acids from their host cells (Daily et al., 2007; Yu et al., 2008; Botte et al., 2013). Genetic studies also demonstrated that FASII is more essential during late liver and mosquito stages (Yu et al., 2008; Vaughan et al., 2009; van Schaijk et al., 2014; Stanway et al., 2019). Quiescent parasites have a larger mitochondrion, closer to the nucleus (Connelly et al., 2021), which retains its function particularly at the level of the respiratory chain (Chen et al., 2014; Peatey et al., 2021). This last point was evidenced by the positive staining of mitochondria in ART-resistant parasites during quiescence (Witkowski et al., 2010; Connelly et al., 2021), and the activity of atovaquone, an inhibitor of the mitochondrial electron transport chain, on quiescent ART-resistant parasites from laboratory strains and Cambodian clinical isolates (Ménard et al., 2015; Reyser et al., 2020; Mok et al., 2021). Such metabolic remodeling in quiescent parasites, which leads to the loss of activity of many antiplasmodial drugs (Reyser et al., 2020, 2023; Ward et al., 2022), makes ART resistance more complex to fight and reinforces the importance of identifying biological targets for the elimination of quiescent parasites.

In the context of the spread of ART resistance worldwide, we wondered whether the apicoplast and the mitochondrion could be therapeutic targets in ART-resistant parasites in the quiescent stage induced by dihydroartemisinin (DHA). We first determined the transcriptome and proteome of ART-resistant parasites upon DHA treatment, and then explored the functioning of fatty acid synthesis (FASII) in relation to glycolysis, and of the mitochondrial electron transport chain, to determine whether these two pathways could be targeted as a countermeasure against ART-resistant parasites in the quiescent stage.

## 2 Materials and methods

### 2.1 Chemicals and drugs

ELQ-271, ELQ-300 and ELQ-400, were kindly provided by Professor Michael K. Riscoe (Portland, USA) and synthetized as already reported (Nilsen et al., 2014). Oligomycin A was purchased from Santa Cruz Biotechnology. Atovaquone (ATQ) was purchased from TCI and 2-deoxy-glucose from Sigma Aldrich. Dihydroartemisinin (DHA) was provided by the WWARN.

### 2.2 *Plasmodium falciparum* cell culture

The ART-resistant *P. falciparum* strain, F32-ART (obtained by drug pressure cycles of increasing artemisinin concentrations and then regularly treated to maintain ART resistance) (Witkowski et al., 2010; Ariey et al., 2014) was cultivated as previously described (Benoit-Vical et al., 2007) at 2% hematocrit in RPMI-1640 medium (GIBCO, France) supplemented with 5% human serum AB. Human red blood cells and serum were obtained from Etablissement Français du Sang (EFS, Toulouse, France). This F32-ART strain has the M476I mutation on the K13 protein giving it its ART resistance profile. Its resistance level is characterized by a mean RSA survival rate of 11% (Ariey et al., 2014; Reyser et al., 2020). Studying the metabolism of quiescent parasites is made difficult by the small number of parasites that survive ART treatment. Cell sorting of quiescent parasites considered to be survivors, only recovers small quantities of biological material, often insufficient for in-depth studies, particularly in transcriptomics and proteomics leading us to conduct the assay on the entire DHA-treated parasite population.

### 2.3 RNA extraction

Synchronized parasites at ring stages were treated for 6 h with 700 nM of DHA or dimethyl sulfoxide (DMSO) as control. Three independent experiments were carried out for each treatment condition. After incubation, drugs were washed away from the cultures with RPMI-1640. Pellets were then washed twice in PBS 1X prior to red blood cell lysis with 0.075% saponin in ice-cold 1X PBS. Extracellular parasites were then washed 3 times in PBS prior to proceeding to RNA extraction according to the manufacturer's instructions (miRNeasy Mini Kit, Qiagen, Hilden, Germany).

### 2.4 Library preparations

Samples quality controls and libraries preparation were performed at GeT-Santé facility (get.genotoul.fr). RNA concentration and purity were determined using a Nanodrop-2000 Spectrophotometer (Thermo Scientific). Integrity of RNA was checked with a Fragment Analyzer (Agilent Technologies France), using the RNA Standard Sensitivity Kit. RNA-seq paired-end libraries were prepared according to the TruSeq Stranded mRNA library prep Kit (Illumina). Libraries quality was assessed using the HS NGS kit on the Fragment Analyzer (Agilent Technologies France).

### 2.5 RNA sequencing

Six libraries were sent for sequencing ((DMSO + DHA) x 3 replicates) at Macrogen Europe. For each sample, 60 million reads were processed. Briefly, sequence raw reads were QC analyzed with FastQC v0.11.7 and trimmed with Trimmomatic 0.38 to remove biases prior the mapping to the *P. falciparum* genome with HISAT2 (v2.1.0), splice-aware aligner. Transcript assembly was performed using StringTie (v2.1.3b). After assembly, the abundance of gene/transcript was calculated as read counts.

Statistical analysis of read counts was produced under R environment (https://www.r-project.org/), using *RStudio* interface (http://www.rstudio.com/). Low-expressed genes were removed from the analysis using the filterByExpr function from the edgeR library. Normalization and differential analysis of the filtered genes were carried out using the DESeq2 library. Genes were considered as differentially expressed between DMSO and DHA treatments when the obtained Bonferroni-corrected *p*-value was <0.05. R codes used for the analysis are available upon request.

RNA-seq raw data are available in the GEO public repository (https://www.ncbi.nlm.nih.gov/geo/query/acc.cgi), accession number GSE207610.

### 2.6 Protein extraction and quantitative proteomics analysis

After incubating the parasites with 700 nM DHA for 6 h, parasite cultures were washed in RPMI-1640. Pellets were then washed twice in PBS before red blood cell lysis with 0.075% saponin (Sigma-Aldrich) in PBS. Parasites ghosts were washed 3 times in PBS before incubating the ghosts with E1A lysis buffer (50 mM Hepes pH 7.5; 250 mM NaCl; 5 mM EDTA; 0.1% Triton X-100, 1 mM DTT, 0.2 mM PMSF supplemented with complete Mini EDTA-free protease inhibitor cocktail (Roche®) for 15 min. Ghosts were then sonicated for 3 × 15 s ON/OFF cycles to shear parasite membranes. Debris were pelleted by centrifuging for 15 min at 12,000 RPM at 4 °C and supernatants (containing parasitic proteins) were then collected. A total of 6 samples were prepared (3 biological replicates for each condition). Protein samples were digested with trypsin according to the manufacturer protocol using S-Trap™ Mini Spin columns (Protifi). Samples were diluted in 5% SDS, 100 mM Tris, pH 8 (135 μL final volume), reduced and alkylated with a solution of 10 mM TCEP, 40 mM chloroacetamide for 5 min at 95 °C, acidified with a final concentration of 1.2% phosphoric acid, diluted with 7 vol of S-Trap buffer (90% MeOH, 100 mM triethylammonium bicarbonate (TEAB), pH 7.1), transferred into S-Trap Mini Spin column, centrifuged at 4000×*g* for 30 s, washed 6 times with S-Trap buffer, digested with 3.3 μg trypsin for 1 h at 47 °C then overnight at 37 °C in 125 μL of 50 mM ammonium bicarbonate buffer. Elution of peptides was performed in 3 steps using first 80 μL of 50 mM ammonium bicarbonate, second 80 μL of 0.2% formic acid, and third 80 μL of 50% acetonitrile/0.2% formic acid.

#### 2.6.1 NanoLC-MS/MS analysis

Peptides were analyzed by nanoLC-MS/MS using a Pro-Flow Nano-Cap-System coupled to a Q-Exactive-HFX mass spectrometer (Thermo Fisher Scientific, Bremen, Germany). Five microliters of each sample were loaded on a C18 precolumn (300 μm ID x 5 mm, Thermo) in a solvent made of 5% acetonitrile and 0.05 % TFA and at a flow rate of 20 μL/min. After 5 min of desalting, the precolumn was switched online with the analytical C18 column (75 μm ID x 50 cm, Pepmap C18, 2 μm particle size) equilibrated in 95% solvent A (5% acetonitrile, 0.2% formic acid) and 5% solvent B (80 % acetonitrile, 0.2 % formic acid). Peptides were eluted using a 5–45% gradient of solvent B over 123 min at a flow rate of 350 nL/min. The Q-Exactive-HFX was operated in a data-dependent acquisition mode with the XCalibur software. Survey scan MS were acquired in the Orbitrap on the 350–1400 *m/z* range with the resolution set at 60000. The 12 most intense ions per survey scan were selected for HCD fragmentation and fragment ions were analyzed in the Orbitrap at a resolution of 15000. Dynamic exclusion was employed within 30 s to prevent repetitive selection of the same peptide. Samples were injected 3 times.

#### 2.6.2 Bioinformatics data analysis of mass spectrometry raw files

Raw MS files were processed with the Mascot software for database search and with Proline (Bouyssié et al., 2020) for label-free quantitative analysis. Data were searched against *Plasmodium falciparum* entries of the UniProtKB protein database. Carbamidomethylation of cysteines was set as a fixed modification, whereas oxidation of methionine and protein N-acetylation were set as variable modifications. Specificity of trypsin/P digestion was set for cleavage after K or R, and two missed trypsin cleavage sites were allowed. The mass tolerance was set to 10 ppm for precursor ions and to 20 mmu in tandem MS mode. Minimum peptide length was set to 7 amino acids, and identification results were further validated in Proline by the target decoy approach using a reverse database at both a PSM and protein false-discovery rate of 1%. After mean of replicate injections, the abundance values were log2 transformed and missing values were replaced by random numbers drawn from a normal distribution using the Perseus toolbox. For statistical analysis, a Student t-test was performed on log2 transformed values to analyse differences in protein abundances in all biological group comparisons. Significance level was set at *p*-value ≤ 0.05. Proteomic data have been deposited to the ProteomeXchange Consortium *via* the PRIDE partner repository with the dataset identifier PXD048354.

### 2.7 Correlation between transcriptomics and proteome analysis

We divided the genes in four groups, depending on whether their expression was differential between treatments on either data set. We then studied the correlation of transcriptomics and proteomics data in each of those groups, and we did so for DHA and DMSO treatments separately. Fig. 2 displays the r2 and the *p*-value of the Pearson Correlation test conducted for each of those groups and treatments.

### 2.8 [13C]-U-glucose labelling and lipidomic analysis

The ART-resistant strain F32-ART was grown in regular culture medium (RPMI-1640 with glucose, 5% human serum, 2.5% hematocrit), and synchronized to ring-stage cultures using D-sorbitol treatment. These ring-stage cultures were then adjusted to a 2.5% parasitemia in RPMI-1640 medium without glucose, 5 % human serum, at 2% hematocrit and divided in two batches to grow either in 8 mM uniformly-labelled [13C] glucose (U-[13C]-glucose) (Eurisotop, Saclay, France) or in 8 mM normal 12C-glucose-complemented medium. Following 42 h of incubation, synchronized ring-stage parasites (at 10 % parasitemia) were treated with 700 nM of DHA for 6 h or with DMSO (at the concentration used as vehicle for DHA) as control. After the 6 h incubation period, the parasites were studied under 2 conditions: (i) at the end of these 6 h, (ii) after a 48-h re-cultivation period. A third condition corresponds to a 24-h DHA treatment in the U-[13C]-glucose or normal 12C-glucose medium. Then the parasite metabolism was rapidly quenched in a dry ice/ethanol bath, either after the 6 h or 24 h DHA treatment period or after 48 h of re-cultivation without treatment but with re-incubation in U-[13C]-glucose or normal 12C-glucose medium for condition 6 h treatment + 48 h of re-cultivation. Parasites were centrifuged at 2500 rpm for 7 min at 4 °C and the parasite pellets were incubated for 10 min in a cold saponin lysis buffer (0.15% saponin, 0.1% Bovine Serum Albumin in PBS 1X). Freed parasites were washed twice with PBS 1X and stored at −80 °C until analysis. Total lipid and aqueous material were extracted from parasites for lipidomic and glucose analysis using Gas chromatography coupled to mass spectrometry (GCMS, Agilent 5977A-7890B) -based lipidomic analyses as previously described (Botte et al., 2013; Amiar et al., 2020). The incorporation of 13C to the fatty acid pool can be determined by the change in mass as an “isotopologue” with mass (M + x, M = original mass from each fatty acid, x = change in the mass by 13C incorporation). Since naturally occurring isotopes of carbon, hydrogen and even oxygen naturally exist in lower abundance, each fatty acid contains masses from M+0 to M+3 in decreasing manner. After subtracting the naturally abundant isotopologues, the 13C incorporation rate was calculated as % 13C incorporation to the total isotopologues. This assay was performed with (U-[13C]-glucose) because only intermediates of glycolysis (PEP, G3DP, DHAP) can be transported into the apicoplast to be used by the FASII pathway and not acetyl-CoA (which could possibly be generated by glutamine and TCA). Labelled glutamine was thus not useful for monitoring FASII in *P*. *falciparum.*

### 2.9 Measurement of *P. falciparum* mitochondrial respiration and glucose consumption

The oxygen consumption rate (OCR) of *P. falciparum* was assessed on the ART-resistant strain F32-ART using the Seahorse XFe24 Extracellular Flux Analyzer (XFe; Seahorse Bioscience, CRCT Toulouse) as described by Sakata-Kato et al. (Sakata-Kato and Wirth, 2016) with minor modifications. Ring-stage parasites were freed from erythrocytes with 50 mL of saponin solution at 0.01% in 1X PBS for 1 mL of infected red blood cells. After 1 min of incubation, the mix was first centrifuged at 800 g for 3 min, and a second time at 3000 g for 6 min for the ring stage or for 3 min for the trophozoite stage. The pellets were washed twice with RPMI-1640 medium at 2500 g for 5 min. Cell density was then adjusted to 12 million cells/100 μL per well for trophozoites and >12 million cells/100 μL per well for rings after microscopic assessment with a hematocytometer. Before seeding freed parasites in the plate, the XF24-well microplate, precoated with CellTak cell and tissue adhesive (Fisher), was washed twice with 1X PBS and once with RPMI-1640. The plates were centrifuged at 50 g for 5 min then 350 μL of RPMI were added to each well; 4 wells were used for background correction with only RPMI and without parasites. The measurement parameters of the assay were configured as follow: the experiment started with the injection of DHA or ATQ, and DMSO used for drugs vehicle was used as control. The OCR and ECAR were measured every 30 min with the following program: mix time 30 s; wait time 90 s; measure time 3 min. For one condition of the experiment, injection of ATQ was made 360 min (6 h) after the DHA injection. For the measurement of mitochondrial respiration fluctuation, we used the Cell MitoStress kit (Agilent, Santa Clara, CA) and the Seahorse XFe24 analyzer according to the manufacturer's instructions with DHA or RPMI in port A, oligomycin A (ATP synthase inhibitor) at 10 μM in port B, FCCP (carbonyl cyanide 4-(trifluoromethoxy) phenylhydrazone, a mitochondrial membrane depolarizer) at 4 μM in port C and 1 μM of a mixture rotenone/antimycin A (antimycin A is a complex III inhibitor at the Qi site) in port D. The glycolytic activity of the parasite responsible for production of lactate was measured through the extracellular acidification rate (ECAR) (Mookerjee et al., 2015), simultaneously to OCR (same culture, wells and time) using to a second fluorescent sensor.

### 2.10 OCR and ECAR statistics

Statistical analysis was carried out under *R* environment (https://www.r-project.org/) using the *RStudio* interface (http://www.rstudio.com/). *Stats* base package was used to run the ANOVA on the mixed models produced by the *nlme* library (Pinheiro et al., 2023). Post-hoc general linear hypothesis tests were carried out with the *multcomp* package (Hothorn et al., 2008), and kinetics breakpoint detection was performed using the *strucchange* library (Zeileis et al., 2002, 2003). Data wrangling and graphic visualization were accomplished with *tidyverse* (Wickham et al., 2019) and *RColorBrewer* (https://rdrr.io/cran/RColorBrewer/).

### 2.11 Evaluation of antiplasmodial activity

SYBR Green I assay was performed to evaluate drug activities (Smilkstein et al., 2004). Parasite cultures, synchronized at the ring-stage by 5% D-sorbitol treatment (Lambros and Vanderberg, 1979), were treated for 48 h with different concentrations of each drug in triplicate at 37 °C and 5% CO_2_. Then, the parasite pellets were washed with 1X PBS and frozen at −20 °C until use. Thawed pellets were incubated with 2X SYBR Green (Thermo Fischer) in lysis buffer (20 mM Tris base pH 7.5, 5 mM EDTA, 0.008 % w/v saponin, 0.08% w/v Triton X-100) for 2 h at room temperature. Fluorescence was measured using a plate reader (FLx800, BioTek) at the wavelength of 485 nm for excitation and 535 nm for emission. The IC_50_ (50% Inhibitory Concentration of parasite growth) values were calculated using GraphPad Prism software 7 (GraphPad Software, San Diego, CA, USA).

### 2.12 Quiescent stage survival assay (QSA)

The drug activity on ART-resistant parasites at the quiescent stage was evaluated on the F32-ART strain by QSA (Reyser et al., 2020). Briefly, D-sorbitol synchronized ring-form culture at 3% parasitemia and 2% hematocrit was first exposed to 6 h of DHA at 700 nM to induce quiescence. Then the parasite cultures were washed with RPMI-1640 medium, resuspended in culture conditions with 5% human serum. The parasites underwent a second treatment for 48 h with DHA at 700 nM, to maintain quiescence, and simultaneously with the molecule to be tested. Different combinations of DHA with the molecule were also tested as controls. At the end of the treatment period, parasite cultures were washed with RPMI-1640 medium and placed in new wells in drug-free culture conditions with 10% human serum. The parasitemia was then monitored daily to determine the time required for each parasite culture to reach the initial parasitemia of 3%. If no recrudescence was observed up to 30 days, the cultures were discarded and the data was censored in the Kaplan-Meier analysis. The dose of 7 μM tested was chosen according to the plasma peak reported in patients for atovaquone and by extension used for ELQ-271, ELQ-300, ELQ-400.

### 2.13 Metabolic pathway enrichment tests

The enrichment of metabolic pathways among differentially expressed genes and proteins was tested using Fisher's exact test under *R* environment (https://www.r-project.org/) using the *RStudio* interface (http://www.rstudio.com/). Proportion of differentially expressed genes/proteins among genes/proteins annotated within a given metabolic pathway was thus compared against the proportion of differentially expressed genes/proteins not annotated within that metabolic pathway.

## 3 Results

### 3.1 Modification of the transcriptomic and proteomic profiles of the ART-resistant strain F32-ART by DHA treatment

**Fig. 1:**
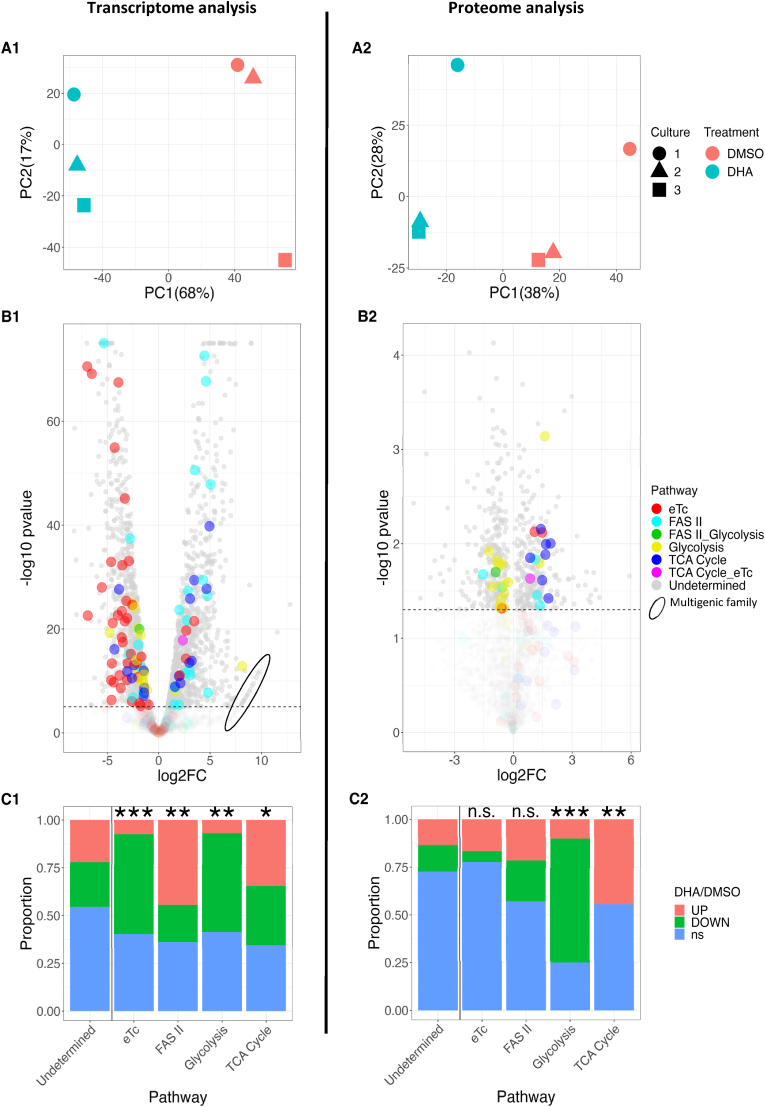
Analysis of transcriptome (left, respectively A1, B1, C1) and proteome (right, respectively A2, B2, C2) of F32-ART ART-resistant parasites after 6 h of 700 nM DHA treatment *versus* DMSO-treated parasites. (**A**) Principal component analysis showing DHA-treated (blue) and DMSO-treated samples (red). Symbol shapes correspond to three independent experiments in the study. Percentages of variance explained by PC1 and PC2 are displayed. (**B**) Volcano plot showing gene or protein log_2_fold-change *versus* -log_10_pvalue. In the transcriptomics plot (B1), the dashed line shows -log_10_pvalue threshold above which Bonferroni correction <0.05 whereas in proteomics plot (B2), the dashed line shows -log_10_pvalue threshold above which the raw p-value is < 0.05. Colors highlight genes or proteins involved in different pathways (Undetermined: genes/proteins involved in as-yet undetermined pathway; eTc: mitochondrial electron transport chain; FASII: fatty acid synthesis II pathway; TCA: tricarboxylic acid cycle). The down-regulated genes/proteins have a negative log_2_fold-change, up-regulated ones have a positive log_2_fold-change. (**C**) Distribution of up-regulated, down-regulated and non-significant genes (C1) (significance: Bonferroni <0.05) and proteins (C2) (significance: raw p-value <0.05) involved in those pathways. The enrichment of metabolic pathways among differentially expressed genes and proteins was tested using Fisher's exact test. Asterisks indicate degrees of statistical significance revealing distribution differences between genes involved, or not, in a given pathway: ∗ (*p*-value <0.1), ∗∗ (<0.01), ∗∗∗(<10^−6^). Distribution of regulated proteins are shown for indicative purposes since *p*-values produced by differential analysis were not adjusted. (For interpretation of the references to colour in this figure legend, the reader is referred to the Web version of this article.)

The impact of DHA treatment on gene expression in ART-resistant parasites was studied using RNA-seq. The first component of the Principal Component Analysis (PCA) of transcriptomic data, accounting for 68% of the total variation of the data, discriminates DHA-treated from DMSO-treated samples. The second component of the PCA, accounting for only 17% of the total variation, accounts for variability between experimental replicates (Fig. 1A1). Comparison of the relative abundance of transcripts in DHA-treated *versus* DMSO-treated ART-resistant parasites revealed that among 4266 detected genes, there was a significant dysregulation of 2201 genes (DEGs, for Differentially Expressed Genes) with 1008 and 1193 genes up- or down-regulated by DHA, respectively (Supplementary file). The volcano plot (Fig. 1B1) provides a visual representation of this differential analysis. As we were interested in FASII and respiration through the electron transport chain (eTc) (Scheme 1), we focused particularly on genes related to these two pathways, but also on those related to glycolysis and the tricarboxylic acid (TCA) cycle, on which FASII and eTc depend. The volcano plot showed that genes involved in those biological pathways were unevenly represented among DEGs, with some pathways appearing globally up-regulated and others, down-regulated (Fig. 1B1). We carried out statistical tests to confirm whether the proportions of up- and down-regulated genes involved in those pathways were significantly different compared to the rest of the genome (Fig. 1C1). The proportion of modulated genes was significantly higher in eTc, glycolysis and FAS II pathways (*p*-values: 7.06 × 10^−07^, 0.002 and 0.009, respectively) (Fig. 1C1). In FASII pathway, however, only three significantly genes coding for key enzymes (FabD, FabG and FabZ), were up-regulated, FabI, B/F and H being unaffected by DHA. Furthermore, genes coding for proteins such as ACP, an essential cofactor for FASII enzyme (Gallagher and Prigge, 2010) were significantly down-regulated (Supplementary file). Moreover, while the synthesis of fatty acids depends on parasite glycolytic activity, half of the genes related to this pathway were under-expressed in DHA- *versus* DMSO-treated parasites. The TCA cycle, which provides electrons to the eTc pathway, was also affected, with 10 genes over-expressed and 10 genes under-expressed out of a total of 28 genes, while 33 out of the 71 eTc-related genes were down-regulated. The volcano plot of fold change of gene expression values revealed an unconventional distribution (encircled on Fig. 1B1, Fig. S1) of a set of genes which mainly belongs to the *rifin* gene family (coding for clonally variant proteins expressed on the surface of the infected red blood cell).

**Fig. 2:**
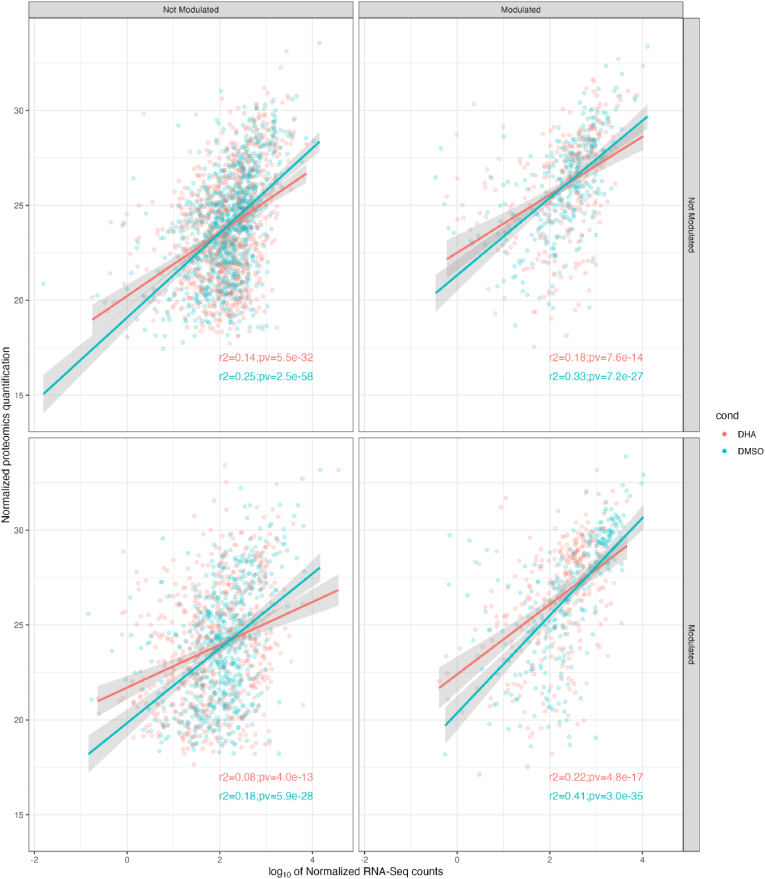
Correlation between normalized transcriptomics (abscises) and proteomics (ordinates) data, for genes/proteins that were detected with both approaches. Each detected gene/protein is represented on only one of the four facets in the figure, according to whether the gene/protein was declared as DHA-modulated, or not, on either approach. Each gene/protein is represented by two circles in the corresponding facet, in blue and red, respectively, corresponding to the mean of the normalized values for that gene/protein on DMSO- and DHA-treated samples. Pearson correlation was tested between transcriptomics and proteomics normalized data for each of those four groups and two conditions. Likewise, linear regression between transcriptomics and proteomics data has been projected on each facet. Coefficient of determination (r^2^) of each linear regression, and *p*-value from Pearson-correlation tests are provided for each group and condition. (For interpretation of the references to colour in this figure legend, the reader is referred to the Web version of this article.)

We then investigated proteomic data on DHA-treated ART-resistant parasites. In this case, the first component of the PCA, also discriminates DHA-from DMSO-treated samples, accounting for 38% of the total variance of the data (Fig. 1A2). The volcano plot (Fig. 1B2) and the distribution of increased and decreased abundance of proteins involved in apicoplast and mitochondrion pathways (Fig. 1C2), also showed a different profile compared to the transcriptomic data.

It is well established that the correlation between transcriptomics and proteomics data is, most often far from optimal. One of the usual reasons, other than issues inherent to each of the approaches, is that the experiments are usually conducted at any given timepoint, when the transcriptional and translational or post-translational studies will necessarily be out of phase with each other. This was indeed the case in our study. That said, we looked at the correlation between the transcriptomics and proteomics normalized data sets (Fig. 2). We reckon it is encouraging to see that the best correlation corresponds to the genes identified here as differential both in transcriptomics and proteomics analysis. However, taken together, these data did not clarify the roles of FASII and eTc pathways in quiescent parasites, and prompted us to conduct a functional approach to evaluate i) FASII implementation within the apicoplast, and ii) the mitochondrial respiration.

### 3.2 Glycolysis shut-down in ART-resistant parasites treated by DHA

**Fig. 3:**
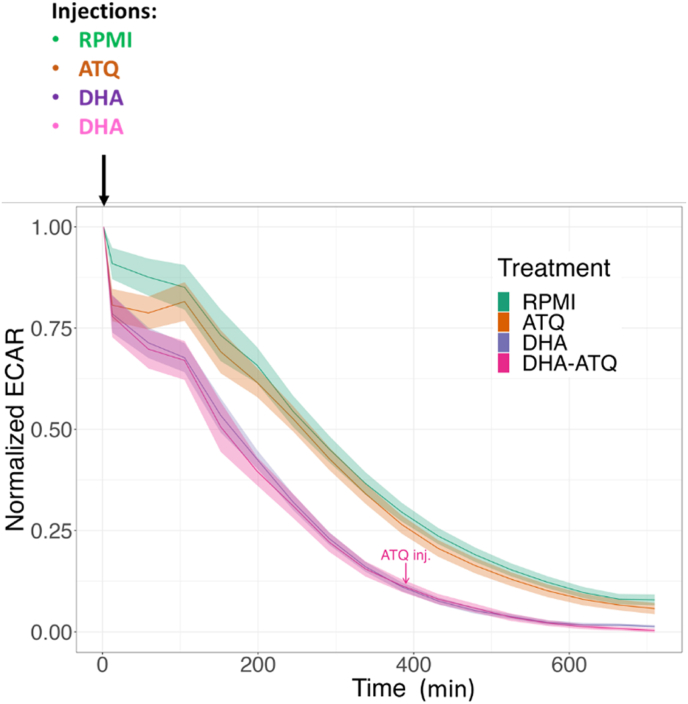
Assessment of the glycolytic activity using Seahorse XFe24 analyzer by measurement of extracellular acidification rate (ECAR) in F32-ART artemisinin-resistant parasites (freed from the host red blood cell) during 720 min (12 h) in presence of 700 nM of DHA or 1 μM ATQ compared to the control RPMI. In the condition DHA + ATQ, DHA was injected alone at the start of the experiment and after 360 min (6 h), ATQ was injected. Data represents the mean ± SEM of 5 independent experiments, each one being the median of several technical replicates. (For interpretation of the references to colour in this figure legend, the reader is referred to the Web version of this article.)

The parasite glycolytic activity (ECAR) was measured at the start of the experiment, as soon as DHA, used to induce quiescence and maintain the ART-resistant parasites in a quiescent state, was injected. We observed a global decrease of ECAR that can be attributed to the experimental extra-erythrocytic condition of the parasites as evidenced in control (RPMI) untreated parasites. However, this decrease was strongly amplified in presence of DHA, whereas atovaquone (ATQ, known to have no effect on glycolysis) had no impact on ECAR compared to untreated parasites nor on DHA-treated parasites (Fig. 3). However, during DHA treatment, it was not possible to determine whether the decrease in glycolysis was attributable to the quiescence state or to the death of parasites, since the use of the control 2-deoxy-D-glucose as glycolysis inhibitor led to highly erratic data (data not shown).

### 3.3 Absence of *de novo* fatty acid synthesis in ART-resistant parasites treated by DHA

The initiation of the prokaryotic type II Fatty Acid Synthesis (FASII) present in *P. falciparum* requires the import and use of phosphoenolpyruvate in the apicoplast, which results from glycolytic activity (Lim et al., 2010; Shears et al., 2015). Therefore, to clarify the possible activation of FASII pathway in ART-resistant parasites during DHA treatment, we used state-of-the-art lipid flux analysis in which we incubated parasites with the stable isotope-labelled U-[^13^C] glucose precursor, and quantified the level of [^13^C] labeling in *de novo* synthesized fatty acid by GC-MS (Botte et al., 2013; Dubois et al., 2018; Amiar et al., 2020). However, since Seahorse analysis showed a down-regulation of glycolysis during DHA exposure (Fig. 3), U-[^13^C] glucose was added before DHA treatment in order to allow the generation of labelled fatty acid precursors prior to cell cycle arrest. U-[^13^C]-glucose uptake in the parasite was confirmed in ART-resistant parasites with detection of labelled glucose among the total glucose isotopologues in both DHA-treated and control parasite aqueous extracts (Fig. S2).

In *Toxoplasma gondii*, a related apicomplexan parasite, the primary fatty acids generated by the apicoplast FASII are lauric acid (C12:0), myristic acid (C14:0), palmitic acid (C16:0) and to a lesser extent stearic acid (C18:0) (Ramakrishnan et al., 2015; Amiar et al., 2016). Therefore, the detection, usually in a “two-by-two” carbon addition, of newly-incorporated ^13^C in any of these usual FASII fatty acids would imply that these fatty acids have been synthesized *de novo via* the apicoplast FASII. If no ^13^C labelled-fatty acids are detected, this would mean that lipids had been salvaged presumably from host lipids.

**Fig. 4:**
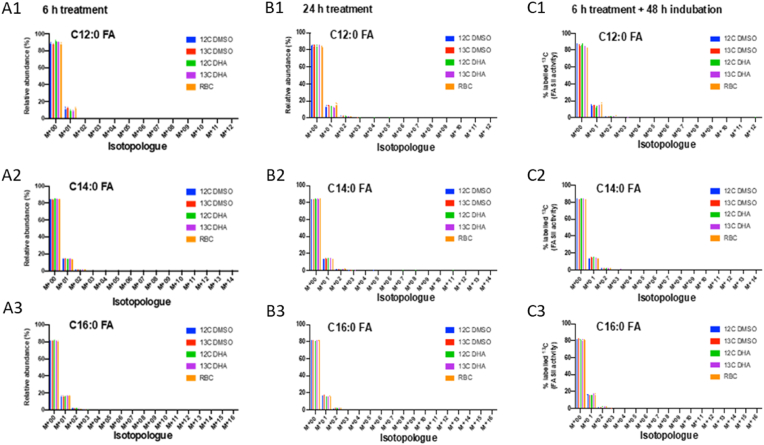
Isotopologue distribution of lauric acid (C12:0), myristic acid (C14:0) and palmitic acid (C16:0) as quantified by GCMS analyses, after 6 h DHA treatment (left panels), 24 h of DHA treatment (middle panels) and 6 h of DHA treatment followed by 48 h of culture (right panels) in F32-ART parasites. M indicates the mass without ^13^C incorporation, and M + x, x indicates the number of ^13^C incorporation. Experiments were performed in 3 independent replicates except for F32-ART 24 h treatment done n = 4 times. Error bar indicates standard error of mean. RBC: non-infected red blood cells. (For interpretation of the references to colour in this figure legend, the reader is referred to the Web version of this article.)

To assess ^13^C incorporation into fatty acids (hence *de novo* synthesis), we analyzed the isotopologues for each of these fatty acids (Fig. 4). As expected, without DHA treatment only naturally abundant isotopologues (M+01, M+02, Fig. 4) of C12:0, C14:0 and C16:0 fatty acids were detected, with no significant U-[^13^C] incorporation (less than 0.1 %), and no difference in parasites incubated either with [^12^C]-glucose or with uninfected red blood cells (RBC) (Fig. 4, Fig. S2, S3). After 6 h of DHA treatment (Fig. 4, Fig. A1, A2, A3), the isotopologue distribution in each fatty acid analyzed was similar to that of DMSO-treated parasites, with no detection of the “two-by-two carbon increase” in the mass of fatty acid isotopologues, which is the signature of FASII activity (Fig. S3A) (Amiar et al., 2020). This could be the result of insufficient time in the 6 h treatment for FASII pathway to be implemented, that is why we reproduced this analysis after 24-h DHA treatment but no additional U-[^13^C]-incorporation into C12:0, C14:0 and C16:0 was detected (Fig. 4, B1, B2, B3). This discrepancy between the up-regulation of some FASII-related genes and the absence of fatty acid *de novo* synthesis could result from the ability of parasites to accumulate transcripts at a given time, to be later translated into proteins (Jackson et al., 2011). To explore this hypothesis, we quantified [^13^C]- incorporation into C12:0, C14:0 and C16:0 in parasites 48 h after the end of a 6-h DHA treatment, but no U-[^13^C]-labelled fatty acids were detected (Fig. 4, C1, C2, C3). Taken together, these results showed no evidence of FASII pathway implementation during DHA treatment, whatever the duration of the treatment, and even after drug removal, suggesting that FASII pathway was not active at the DHA-induced quiescent stage, nor in recrudescent parasites after DHA treatment.

To determine whether DHA treatment had an impact on parasite lipid/fatty acid homeostasis, which could illustrate changes in lipid acquisition during DHA treatment, we conducted whole lipid and fatty acid analyses by GCMS approach for each treatment condition. The only detectable change in the fatty acid composition for the ART-resistant parasites (Fig. S4) immediately after 6 h exposure to DHA, was a reduction in C14:0, whereas 24 h DHA treatment led to an increased abundance of C12:0 and a decrease in eicosenoic acid (C20:1). By contrast, in parasites re-cultivated 48 h in DHA-free medium after a 6 h DHA treatment, no variation was observed, compared to the non-treated parasites (Fig. S4).

Therefore, we did not detect any *de novo* synthesis of fatty acids in ART-resistant parasites upon DHA exposure. DHA treatment also had little impact on the lipid profile of ART-resistant parasites, with fatty acid composition remaining almost unchanged.

### 3.4 Evidence of mitochondrial respiration capacity in ART-resistant parasites treated by DHA

**Fig. 5:**
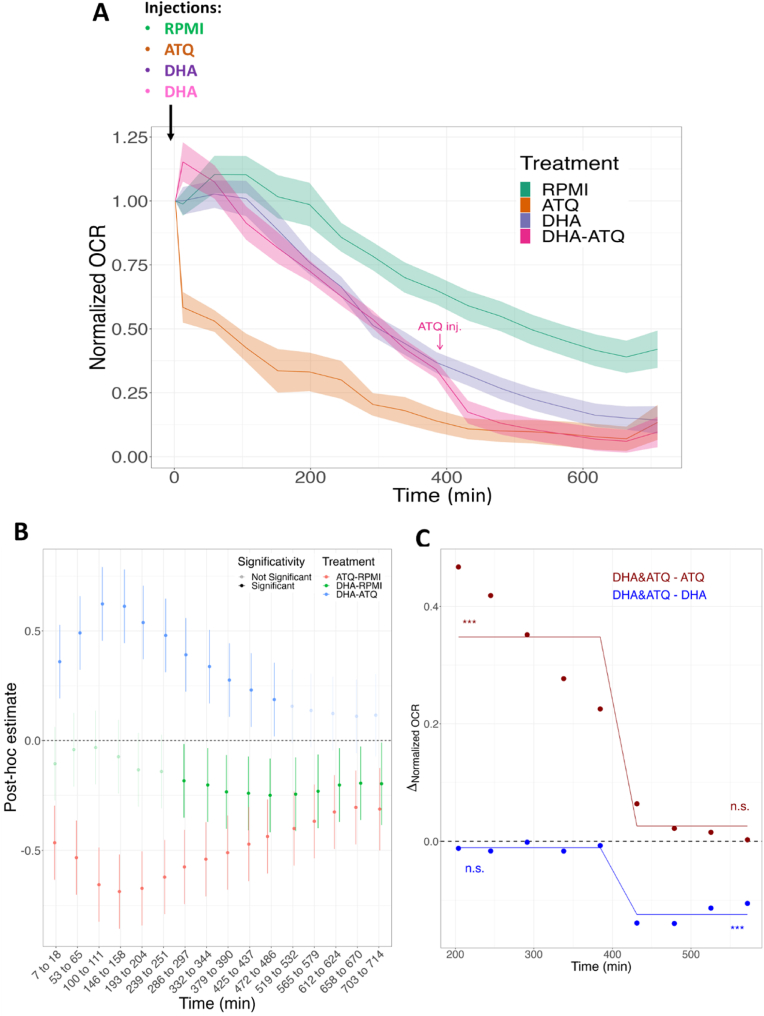
Measurement of mitochondrial respiration using Seahorse XFe24 analyzer in F32-ART ART-resistant parasites. (**A**) Kinetic of oxygen consumption rate (OCR) readouts during 720 min (12 h) of control RPMI, DHA (700 nM) and ATQ (1 μM) treatments. In the condition DHA + ATQ, DHA was injected alone at the start of the experiment and after 360 min (6 h), ATQ was injected. Data represents the mean of 5–7 experiments ± SEM. (**B**) General Linear Hypothesis post-hoc test. Data represent mean differences between two treatments ± CI (95%) at a given time frame. Semi-transparent differences are considered as non-significant (adjusted *p*-value <0.05), *i.e*. when the absence of difference (dashed line) is comprised within the CI. (**C**) Computation of breakpoints and fitted dynamics in OCR readout before and after the addition of ATQ at 360 min. Dots represent the differences, in the 200–600 min timeframe, between normalized OCR readouts in the DHA samples where ATQ was added, and plain ATQ (red) or DHA (blue) readouts. A single breakpoint was detected in each of those two series, corresponding to the addition of ATQ. Lines depict fitted differences after breakpoint computation. DHA + ATQ readouts were significantly different from DHA readouts after the addition of ATQ (∗∗∗: *p*-value <10^−6^), and non-significantly different (n.s.) to ATQ readouts. The opposite was true before the addition of ATQ. (For interpretation of the references to colour in this figure legend, the reader is referred to the Web version of this article.)

The mitochondrial respiration of parasites was investigated during DHA treatment by assessing the modulation of the Oxygen Consumption Rate (OCR) with a Seahorse XFe24 Extracellular Flux Analyzer, based on prior work done on *P. falciparum* and *Trypanosoma cruzi* (Sakata-Kato and Wirth, 2016; Gonzalez-Ortiz et al., 2019). We previously demonstrated by pharmacological approaches that atovaquone (ATQ), an eTc inhibitor, was able to impair the recovery of ART-resistant parasites maintained in a quiescent state under DHA treatment (Reyser et al., 2020). Here we functionally explored the effect of ATQ on the respiration of these persistent parasites. The OCR of saponin-freed ring-stages of ART-resistant parasites was quantified for 720 min (12 h), the longest monitoring period provided by the Seahorse instrument, in DHA- and/or ATQ-treated samples, as well as in RPMI, untreated parasites (Fig. 5).

A gradual decline of OCR was observed throughout the experiment duration, regardless of the condition, likely due to the fragility of ring-stage parasites outside the red blood cells (RPMI control condition, Fig. 5A). An injection of ATQ alone at the beginning of the experiment caused, as expected, a strong collapse of the OCR in a few minutes time, while DHA effect was visible only after a few hours. When ATQ was added 6 h after DHA injection, a rapid collapse of the OCR was also observed (Fig. 5A and B). Time series OCR stability of the double treatment DHA/DHA + ATQ (namely DHA&ATQ) was statistically analyzed, and compared to the individual treatments, DHA or ATQ. One single breakpoint was revealed as statistically significant in DHA&ATQ samples between 200 and 600 min, corresponding to the injection of ATQ at ∼400 min (Fig. 5C). Before the addition of ATQ, the difference between the DHA&ATQ and DHA was, as expected, non-significant, since ATQ was not yet introduced. After the addition of ATQ and the subsequent breakpoint in time series, the difference between DHA&ATQ and DHA was henceforth statistically significant. By contrast, the values of OCR from the wells DHA&ATQ and ATQ were significantly different before ATQ addition, while those differences were non-significant after the addition of ATQ (Fig. 5B). Clearly, despite the inevitable progressive decrease of OCR signal during the experiment, a functional respiratory chain was revealed during DHA treatment thanks to the ATQ injection, suggesting cellular respiration in DHA-induced quiescent parasites.

**Fig. 6:**
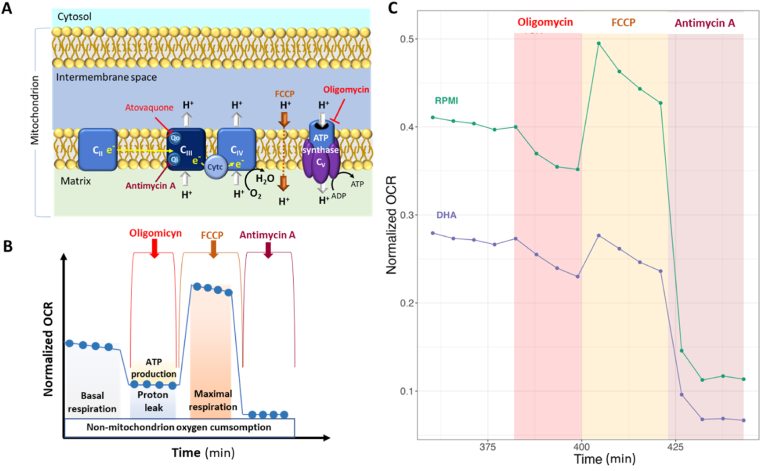
**Measurement of mitochondrial fluctuation of the respiration using Seahorse XFe24 analyzer in F32-ART artemisinin-resistant parasites. (A, B)** Principle of the mitochondrial stress assay at different levels of the electron transport chain (based on Agilent Seahorse XF Cell Mito Stress test Kit, User guide). **(C)** Time course and live bioenergetic profile after sequential addition of oligomycin A (10 μM), FCCP (4 μM) and antimycin A (1 μM). Each point of the curves represents the mean of 7 technical replicates of one experiment.

Moreover, we showed that the OCR can be modulated on saponin-freed ring-stage parasites both on untreated (control RPMI) and on DHA-treated parasites, even after 6 h of treatment, by the sequential addition of different reagents (method from Agilent Seahorse XF Cell Mito Stress Test Kit, Kit 103015-100, User Guide, 2019) (Fig. 6A and B). Oligomycin A, a complex V inhibitor, was firstly injected as indicator of the ATP-linked production and proton leak respiration, and led to a decrease of the basal respiration in untreated parasites, but also in DHA-treated parasites. FCCP, an uncoupling agent of mitochondrial oxidative phosphorylation, was then added to collapse the inner membrane proton gradient. FCCP led to a restoration of a respiratory capacity of the electron transport chain in untreated parasites, but also in DHA-treated parasites, albeit to a lesser extent. Finally, antimycin A, a complex III inhibitor, was added last to shut-down the electron transport chain, which led to a very strong decrease of the OCR in both untreated and DHA-treated parasites (Fig. 6C). All these data showed a lower level of respiration in the DHA-treated parasite population comparatively to untreated one, but the maintenance of a certain functional integrity of the mitochondrion and its electron transport chain in DHA-induced quiescent parasites.

### 3.5 Elimination of *P. falciparum* ART-resistant parasites at the quiescent stage by targeting mitochondria

The present evidence of the functionality of mitochondrial respiration in DHA-treated parasites and the possibility of recovery for quiescent parasites using the eTc inhibitor ATQ (Reyser et al., 2020), led us to explore the activity of three Endochin-like quinolones (ELQ), potent inhibitors of cytochrome *bc*_1_ complex, in quiescent ART-resistant parasites. Chemosensitivity assay of ELQ-271, ELQ-300 and ELQ-400, known to present a strong antiplasmodial activity in the low nanomolar range (Nilsen et al., 2014; Song et al., 2018), showed that ELQ-400 was the most active, presenting an IC_50_ value of 3 nM, similar to the value obtained with ATQ or DHA (Table 1).

**Table 1:** Antiplasmodial activity of mitochondrial inhibitors against *P. falciparum* F32-ART strain.

Drugs	Chemical structures	Mean IC_50_ ± SEM (nM)
Atovaquone		1.5 ± 0.4
ELQ-271		40 ± 13
ELQ-300		14 ± 4.5
ELQ-400		3 ± 0.9
DHA		2.5 ± 0.9

Data represents mean of at least three independent experiments. DHA was routinely tested, as antiplasmodial control drug.

**Fig. 7:**
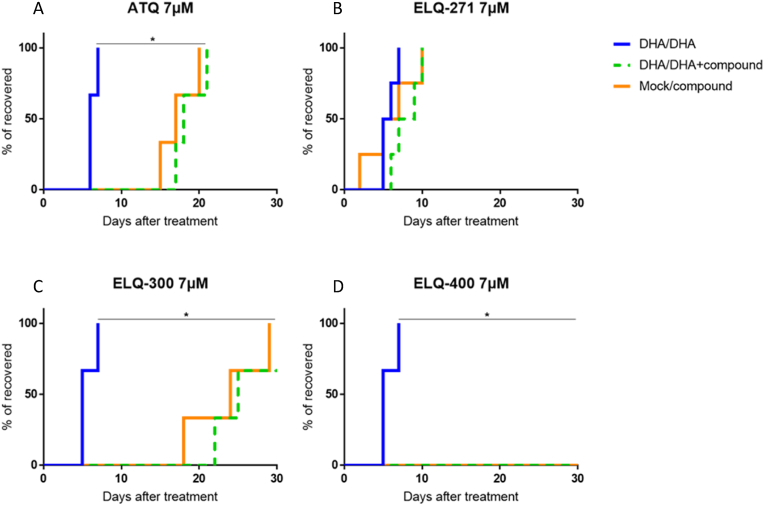
Evaluation of the activity of three ELQ towards F32-ART parasites in the Quiescent stage survival assay (QSA) after a 48-h exposure. Kaplan-Meier survival curves showing recrudescence days after different drug treatments (DHA at 700 nM and other compounds at 7 μM regarding the plasma peak of atovaquone (Nixon et al., 2013a). When appropriate, statistical significance was ascertained by using a log-rank (Mantel-Cox) test. Analysis of at least 3 independent experiments.

We also demonstrated the excellent efficacy of the ELQ compounds in the Quiescent-stage Survival Assay (QSA) (Reyser et al., 2020) (Fig. 7). After exposure to both DHA and ELQ, the parasite recrudescence was followed over a maximum of 4 weeks. A delay in recrudescence time above 6 days between the conditions “DHA 6 h/(DHA + molecule) 48 h” and “DHA 6 h/DHA 48 h” was considered as relevant of activity of the tested molecule against quiescent parasites (Reyser et al., 2020). Atovaquone, inhibiting the cytochrome *bc*_1_ complex (also named complex III) of the mitochondrial respiratory chain (Siregar et al., 2015), was used in QSA as positive control since its activity against quiescent parasites was already reported (Chen et al., 2014; Peatey et al., 2015; Reyser et al., 2020). ATQ activity against ART-resistant DHA-treated parasites was here confirmed with an extended delay of 12 days in recrudescence time (Fig. 7) comparatively to DHA 48 h treatment. ELQ-300 and ELQ-400, tested at the same dose of 7 μM as atovaquone (Fig. 7A) according to the atovaquone plasma peak concentration in patients (Nixon et al., 2013a), were also highly active in QSA conditions. ELQ-300 treatment on quiescent parasites led to a recrudescence time of more than 20 days (Fig. 7C), and no parasite recrudescence was observed within 30 days after treatment with ELQ-400 (Fig. 7D). ELQ-271 was inactive in these conditions (Fig. 7B), despite its IC_50_ value at the nanomolar range. This pharmacological approach confirmed the OCR data that showed a significant and flexible mitochondrial activity of ART-resistant parasites under DHA treatment, and clearly demonstrated that eTc can be targeted to eliminate ART-resistant parasites at the quiescent stage.

## 4 Discussion

In the current context of ART resistance and with the goal of identifying therapeutic targets to support the malaria drug discovery process, apicoplast and mitochondrion metabolisms were studied in ART-resistant parasites during the quiescent stage induced by exposure to DHA, and during the subsequent parasite recovery period. As previously reported, the FASII pathway is flexible and can be turned on or off depending on the parasite's needs (Yu et al., 2008; Vaughan et al., 2009; van Schaijk et al., 2014; Stanway et al., 2019). Here, the question was whether the FASII pathway may be activated during ART-induced quiescence. Our transcriptomic results measured just after the 6 h of DHA exposure suggested an activation of some key enzymes of the FASII system, especially FabD, FabG, and FabZ. Moreover, some other enzymes remained at a very low basal level, such as FabB/F, FabH and FabI. The ACP gene, encoding another key element of the FASII pathway, was found down-regulated (Fig. 1 B1, 1C1). However, our proteomic data did not show significant implementation of any protein involved in the FASII pathway (Fig. 1C2). It is to note that the samples for both gene expression and protein quantification were harvested at the same time whereas globally 11 h are necessary to obtain a protein peak abundance (Foth et al., 2011) from gene expression, which could explain the discrepancy between the FASII enzymes transcriptomic and proteomic data. We then conducted a functional study of the FASII pathway activity by measuring *de novo* fatty acid synthesis and the lipidic composition of the parasites during DHA treatment. The absence of ^13^C labelled fatty acids as lauric acid (C12:0), myristic acid (C14:0), palmitic acid (C16:0) and to a lesser extent stearic acid (C18:0), in control condition (DMSO-treated) confirmed previous studies describing the lack of *de novo* biosynthesis of fatty acids in proliferating parasites in the erythrocytic stage under regular *in vitro* growth conditions (Yu et al., 2008) (Fig. 4). When parasites are quiescent, regardless of the DHA treatment duration (6–24 h), no U-[^13^C]-labelled fatty acids were detected suggesting again the absence of *de novo* synthesis of fatty acids, and thus the absence of a functional FASII pathway, in quiescent parasites when using glucose as a carbon source. As no newly synthetized fatty acids were detected up to 48 h after the end of the DHA treatment, the FASII pathway does not appear to be involved in the parasite awakening phase either. Our results evidencing the absence of FASII pathway activity in quiescence induced by DHA are consistent with previous data showing that when parasites are grown in a minimum lipid medium, FASII pathway is not activated to generate more fatty acids necessary for their development (Botte et al., 2013; Amiar et al., 2020). Moreover it has been also shown that the parasite constitutively scavenges massive amounts of fatty acids from host RBC lipids, notably through the lysophospholipase LPL3 (Sheokand et al., 2023). These scavenged fatty acids are then stored in parasite lipid droplets, and mobilized to allow parasite division (Dass et al., 2021; Sheokand et al., 2023), which could make them immediately available when parasites emerge from quiescence. Nothing is known about the impact of artemisinins treatment on the import of host fatty acids. Although a FASII-type metabolism was not detected in quiescent parasites, some changes in the contents of various fatty acids were observed following exposure of the parasites to DHA. These modifications in the parasite fatty acid content could be explained by a change in the uptake of the fatty acid source from the host red blood cell and culture medium (human serum) and alteration of the parasite's membranes in response to DHA treatment (Fig. S4). However, our data showed that DHA treatment had little effect on the lipid homeostasis on the ART-resistant parasites with a fatty acid composition almost not modified indicating the maintenance of the lipid homeostasis in ART-resistant quiescent parasites. Interestingly, the few modifications observed in lipid homeostasis for the ART-resistant strain could be a sign of a certain preservation of the structural integrity of the parasites despite DHA treatment, as already demonstrated (Egwu et al., 2022). Thus, in the DHA-treated parasites, the lack of incorporation of ^13^C from glucose into fatty acids and the profile of fatty acid, similar to those of DMSO-treated parasites, highlighted that the FASII pathway was not a good target to eliminate quiescent parasites.

As glycolysis is considered to be the main source of energy in asexual stages and of carbone for fatty acid biosynthesis (Cobbold and McConville, 2014), we used ECAR measurement, as an indicator of the glycolytic and respiratory activities in ART-resistant parasites exposed to DHA. The ECAR data showed a decrease in glycolytic activity in line with the transcriptomic data, in which half of the glycolytic genes are repressed (Fig. 1C1, Fig. 3). Although the death of a part of the parasites during DHA treatment as a reason for the decrease in glycolytic activity cannot be excluded, our data support previous studies showing that glycolysis is reduced in quiescent parasites (Chen et al., 2014; Mok et al., 2021) and thus provides a limited source of FASII precursors during quiescence. While many down-regulated genes are linked to mitochondrial pathways (Fig. 1), quiescent parasites have been shown to retain mitochondrial respiration, as evidenced by fluorescent staining (Chen et al., 2014; Peatey et al., 2015), even after 48 h of ART exposure (Witkowski et al., 2010). Moreover, the measurement of mitochondrial respiration using a Seahorse XFe24 analyzer in ART-resistant parasites demonstrated a functional mitochondrion in quiescent parasites. Despite the accumulation of constraints unfavorable to the survival of parasites outside the red blood cells, we evidenced that persistent parasites, even after 6 h of DHA-treatment, have an atovaquone sensitive and functional mitochondrion that is flexible as demonstrated with the sequential addition of different reagents, each targeting specifically an element of the mitochondrial electron transport chain (eTc) (Figs. 5 and 6). Oligomycin A led to a slight decrease of OCR that reflects a weak mitochondrial ATP synthesis inhibition in both proliferative (DMSO-treated) and quiescent (DHA-treated) parasites. FCCP injection also restored mitochondrial respiration in both proliferative and quiescent parasites. The absence of a higher FCCP response for quiescent parasites than for DMSO-treated reflected the lack of spare respiratory capacity, suggesting that during quiescence mitochondrial respiration is operating at its full capacity (Fig. 6). Antimycin A injection after FCCP resulted in a very strong decrease of the OCR in both cases, DHA-treated and untreated. Interestingly, all these data, showing a functional respiratory chain in DHA-induced quiescent parasites, may explain why they are especially sensitive to the ATQ inhibitor of the Qo site of the cytochrome *bc*_1_ complex. Therefore, eTc in *P. falciparum* mitochondrion involved in the development and survival of the intraerythrocytic parasite appears to be a relevant target to eliminate quiescent parasites induced by DHA. The eTc is composed of four enzymatic complexes representing as many potential targets. To date, the eTc complex III, also known as cytochrome *bc*_1_ oxidase, is the only target of known antimalarial drugs such as atovaquone (Nixon et al., 2013b; Siregar et al., 2015) which acts as ubiquinone competitor for the quinone oxidation (Qo) site of the cytochrome *bc*_1_ complex (Stickles et al., 2015). However, the use of ATQ in the field results in a rapid emergence of resistance due to mutations in the Qo domain (Vaidya and Mather, 2000). New complex III inhibitors belonging to 4(1H)-quinolone-3-diarylethers antimalarial class, ELQ-400, ELQ-300 and ELQ-271 (Nilsen et al., 2013; Song et al., 2018), were recently reported to be active on atovaquone-resistant strains thanks to their efficacy against another active site of the complex III, the quinone reduction (Qi) functional site. ELQ-400 due to its unique mode of action, primarily on the Qo but also on the Qi sites, was found to be more active than ELQ-300, and not active on the Qo site (Stickles et al., 2016; Song et al., 2018). Despite its IC_50_ in the nanomolar range (Table 1), ELQ-271 which only targets the Qi site (Song et al., 2018) was not active in the QSA, similar to what is observed with DHA (Fig. 7). By contrast, ELQ-300 and ELQ-400 exhibited high activity on ART-resistant quiescent parasites. This is particularly interesting given that of the artemisinin partner drugs in ACTs, only lumefantrine and mefloquine have shown activity against quiescent parasites in QSA (Reyser et al., 2020). These results were in accordance with those obtained with atovaquone in OCR measurement and confirmed that the mitochondrial electron transport chain represents an excellent target to eliminate both proliferating and ART-resistant quiescent parasites. Taken together our results suggest that ELQ-300 and ELQ-400 could be considered as partner drugs in ACTs as a new class of anti-eTc, capable of eliminating both proliferative and quiescent parasites.

## 5 Conclusion

In the present study, thanks to U-[^13^C]-glucose labeling studies, our findings suggested that there is no metabolic evidence of *de novo* fatty acid synthesis in ART-resistant parasites at both proliferating and DHA-induced quiescent states. Therefore, FASII pathway did not seem to be a relevant target for the elimination of ART-resistant parasites when they are quiescent. Conversely, the active metabolic status of the mitochondrial electron transport chain in both proliferative and quiescent ART-resistant parasites confirmed the high interest for this pathway. Interestingly, novel compounds as ELQ targeting eTc appeared to be promising tools to eliminate parasites in the alarming ART resistance context.

## Data availability

Transcriptomic data are available in the GEO repository (https://www.ncbi.nlm.nih.gov/geo/) with the accession number GSE207610. Proteomic data are available in the PRIDE repository (http://www.ebi.ac.uk/pride) with the accession number pxd048354.

## CRediT authorship contribution statement

**Manel Ouji:** Writing – original draft, Methodology, Conceptualization. **Thibaud Reyser:** Writing – original draft, Methodology. **Yoshiki Yamaryo-Botté:** Writing – original draft, Methodology, Data curation. **Michel Nguyen:** Writing – review & editing, Methodology. **David Rengel:** Writing – review & editing, Software, Methodology, Investigation, Conceptualization. **Axelle Dutreuil:** Methodology. **Marlène Marcellin:** Writing – review & editing, Methodology. **Odile Burlet-Schiltz:** Writing – review & editing, Methodology. **Jean-Michel Augereau:** Writing – review & editing, Writing – original draft, Validation, Supervision, Formal analysis, Data curation, Conceptualization. **Michael K. Riscoe:** Writing – review & editing, Investigation, Formal analysis. **Lucie Paloque:** Writing – review & editing, Writing – original draft, Visualization, Validation, Supervision, Formal analysis, Data curation, Conceptualization. **Cyrille Botté:** Writing – review & editing, Writing – original draft, Investigation, Funding acquisition, Formal analysis. **Françoise Benoit-Vical:** Writing – review & editing, Writing – original draft, Validation, Supervision, Project administration, Funding acquisition, Formal analysis, Data curation, Conceptualization.

## Declaration of competing interest

The authors declare no competing interests.
