# Supplementary material for: In artemisinin-resistant falciparum malaria parasites, mitochondrial metabolic pathways are essential for survival but not those of apicoplast
Source: Int J Parasitol Drugs Drug Resist. 2024 Sep 19;26:100565. doi: 10.1016/j.ijpddr.2024.100565 (PMC11466614; doi:10.1016/j.ijpddr.2024.100565)
Supplement: Multimedia component 2 [file mmc2.docx]

*
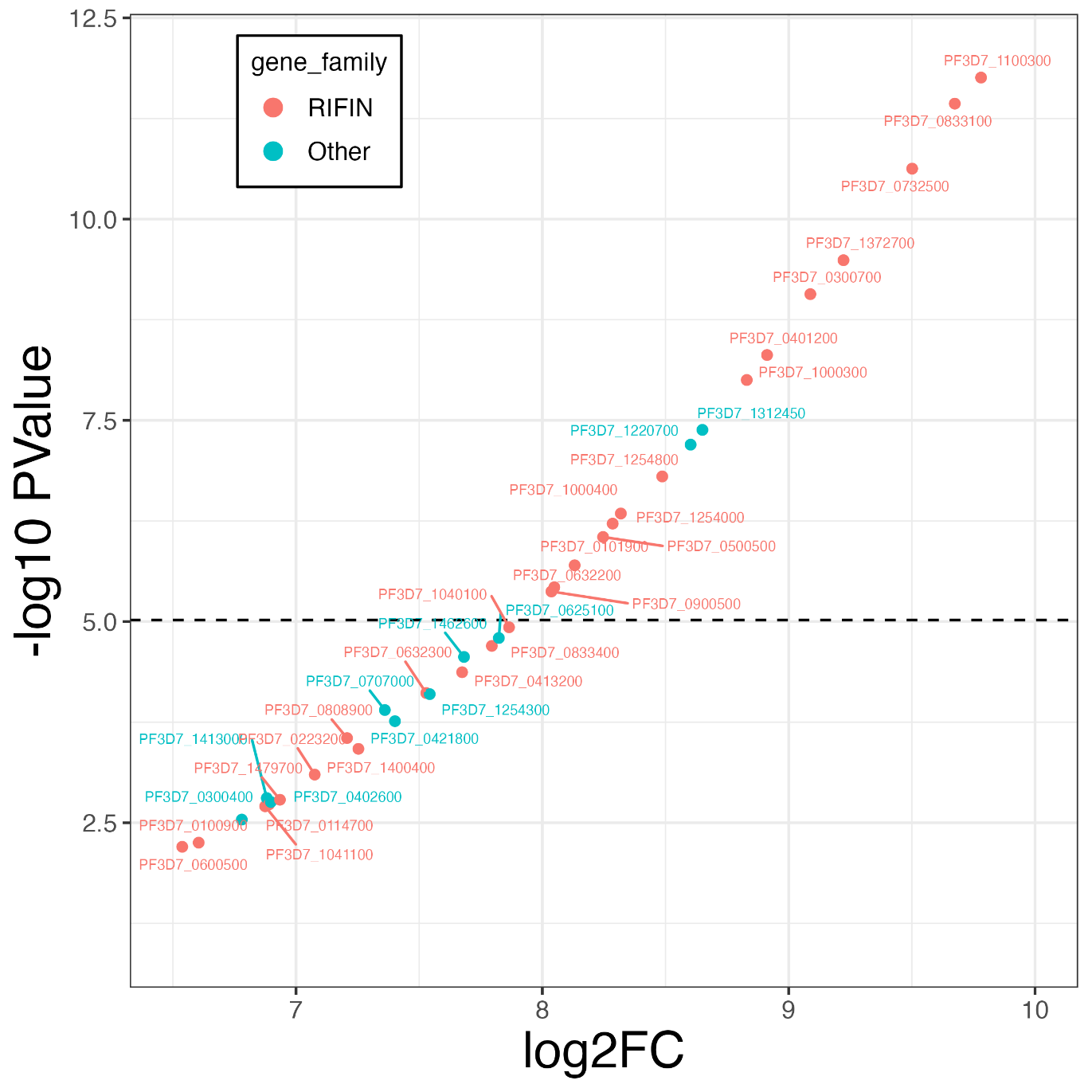
*

**Figure S1**: Detail of the multigenic family highlighted on the volcano plot (Fig. 1 B1) from transcriptomic comparison of artemisinin-resistant parasites F32-ART after 6 hours of 700 nM DHA treatment *versus* untreated parasites. The dashed line shows -log_10_pvalue threshold above which Bonferroni correction < 0.05.

**
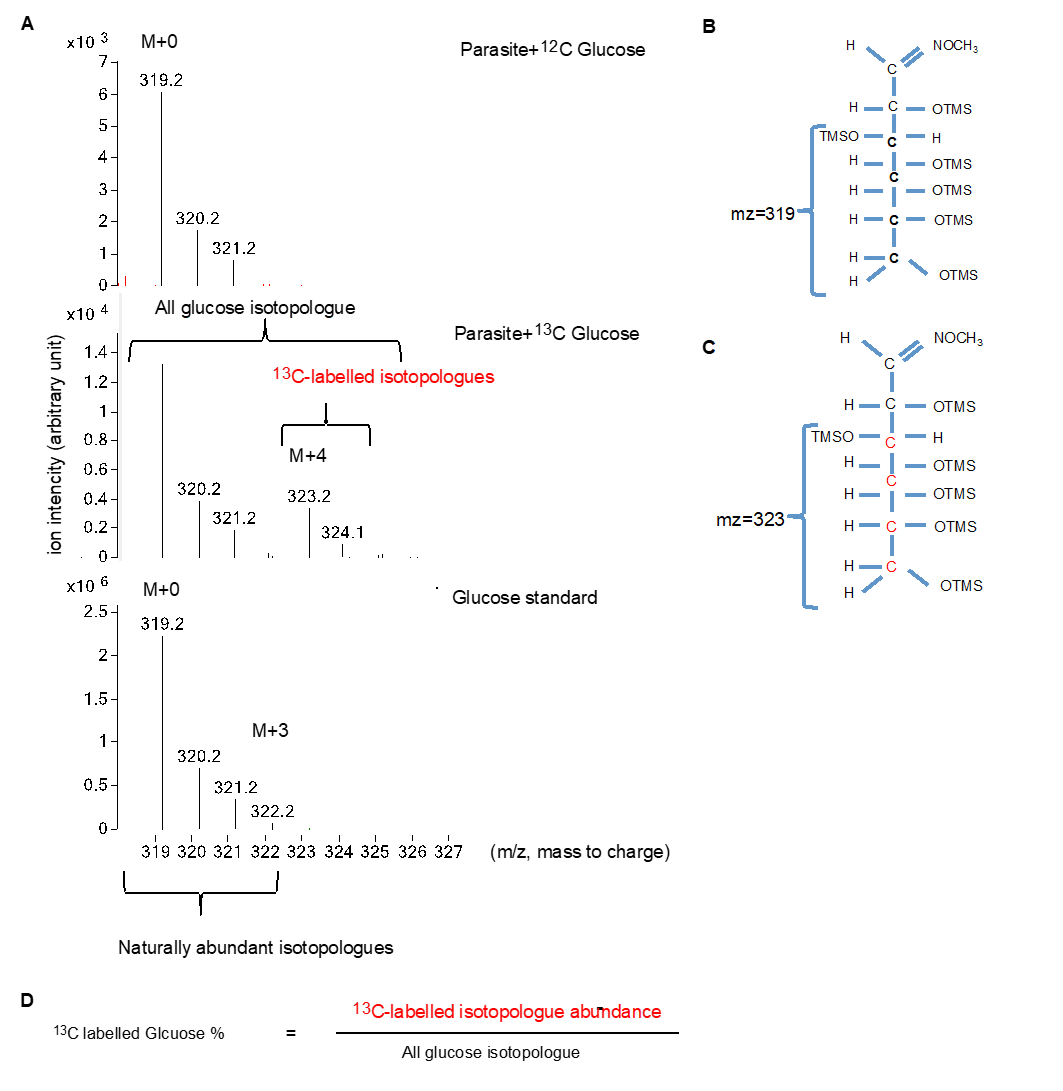
**

**Figure S2: (A)** Chromatogram depicting the representative fragmentation glucose from ^12^C glucose incorporated parasite, ^13^C glucose incorporated parasite and ^12^C glucose standard as determined by GCMS. (**B)**, **(C)** Fragmentation patterns for ^12^C glucose and ^13^C glucose with specific fragment mass of m/z=319 and 323, respectively**. (D)** ^13^C labeled glucose % was calculated as shown above for the myristic acid calculation.

**
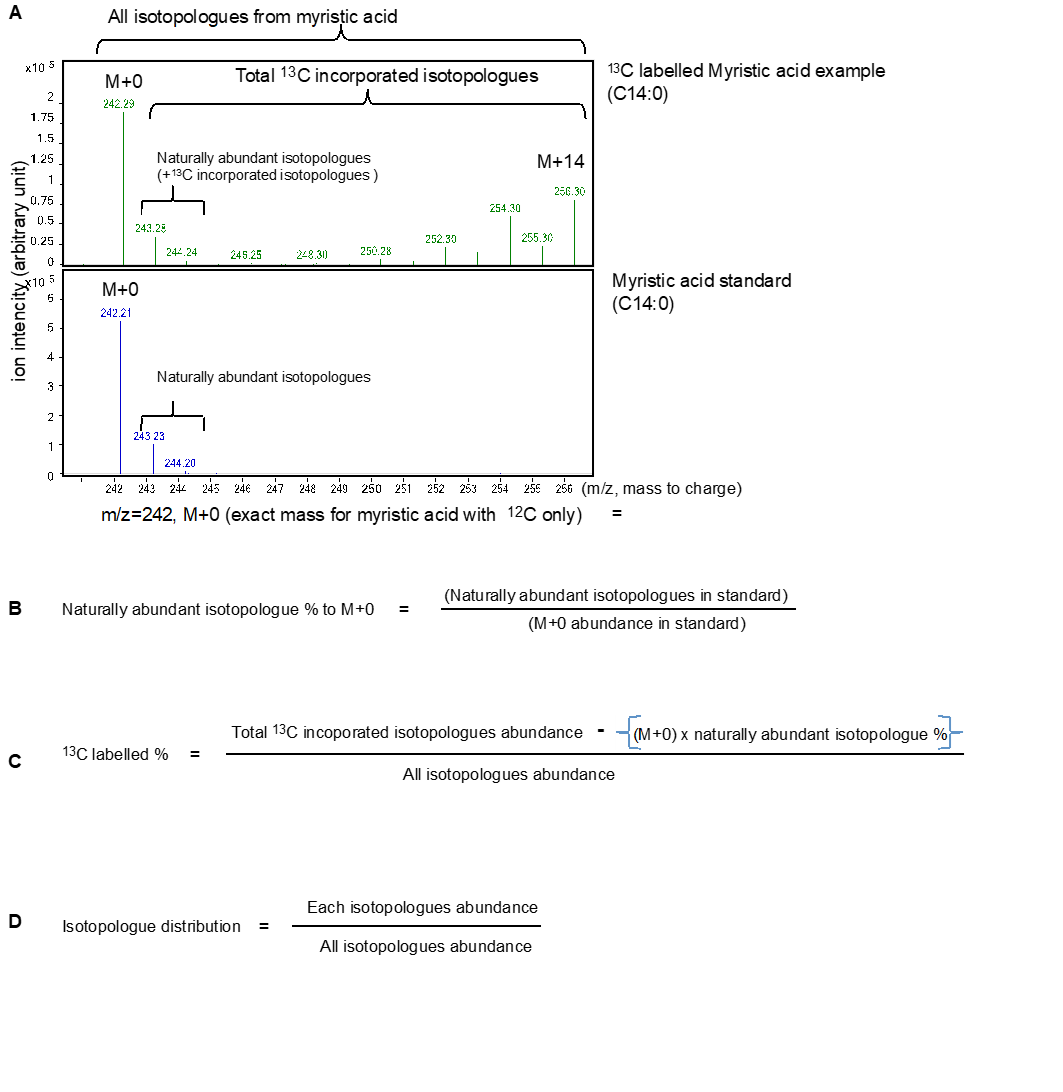
**

**Figure S3:** (**A**) Example of a GCMS chromatogram illustrating ^13^C incorporation to myristic acid (C14:0) and standard myristic acid fragmentation (Adapted from Phillips, 2015). (**B)** The naturally abundant isotopologue % was calculated from the standard isotopologue abundance. (**C)** ^13^C labelled % presented in the Figure 4 was calculated according to the equation. (**D)** Isotopologue distribution was calculated and shown in graphical form in the Figure 4.

**
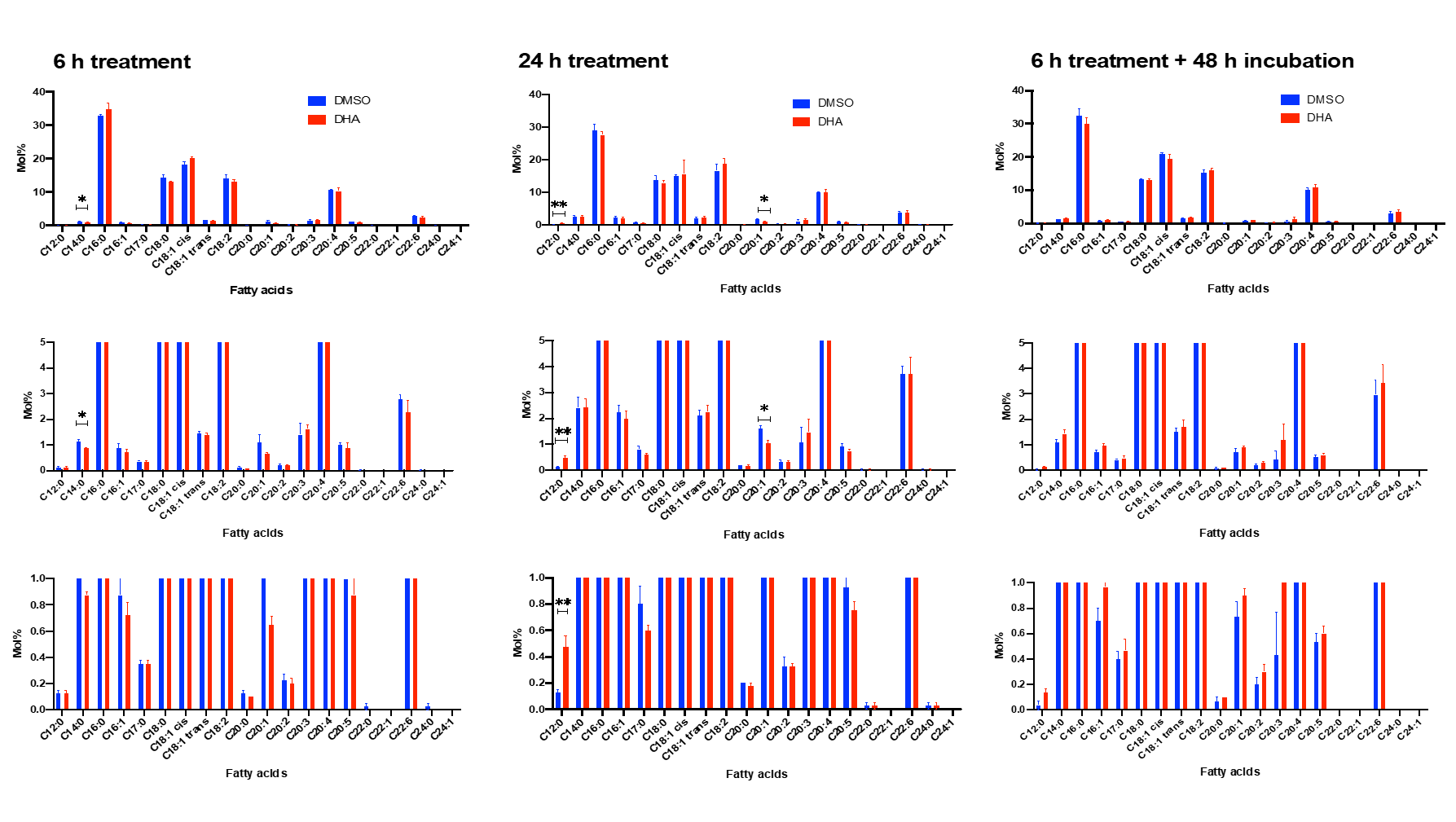
Figure S4. Fatty acid composition** (relative fatty acid molar abundance) **in F32-ART** in 6 h DHA treatment (left panels), 24 h of DHA treatment (middle panels) and 6 h of DHA treatment followed by 48 h of incubation without treatment (right panels). The second and third lines correspond to a zoom of the first line. Experiments performed in 3 independent assays except for 24 h treatment done 4 times. Error bar indicates standard error of mean. The star indicates the *p-*values **p* < 0.05; ***p* < 0.01
